# Intraperitoneal perforation through lymph node metastases in a patient with esophageal squamous cell carcinoma during intensive chemotherapy: A case report with literature review

**DOI:** 10.1016/j.ijscr.2022.106944

**Published:** 2022-03-22

**Authors:** Tomonari Suetsugu, Yoshihiro Tanaka, Yuta Sato, Masahiro Fukada, Itaru Yasufuku, Kazuhiro Yoshida

**Affiliations:** Department of Gastroenterological Surgery, Gifu University Graduate School of Medicine, Gifu City, Japan

**Keywords:** Biweekly DCF therapy, Chemotherapy, Esophageal cancer, Triplet regimen

## Abstract

**Introduction:**

Esophageal fistula after treatment is a critical and fatal complication of esophageal cancer. A fistula forming from lower thoracic esophageal cancer to the peritoneum through lymph node metastases following chemotherapy has not been reported. We report a case of peritonitis due to lymph node perforation through the tumor ulcer after induction of biweekly docetaxel, cisplatin, and 5FU combined chemotherapy (Bi-DCF) for advanced esophageal squamous cell carcinoma (ESCC).

**Presentation of case:**

A 48-year-old woman was referred to us with a diagnosis of lower thoracic ESCC and thoracoabdominal aortic aneurysm. Esophagogastroduodenoscopy showed a circumferential type 3 tumor with stenosis in the lower thoracic esophagus. Contrast-enhanced computed tomography (CT) showed a thoracoabdominal aortic aneurysm and wall thickening of the lower thoracic esophagus that was suspicious of esophageal cancer. Lymph node metastases dumpling from around the tumor to abdominal cavity were also observed. The initial diagnosis was ESCC T3 N3 M1 (para-aortic lymph nodes and liver) Stage IVB. She was started on Bi-DCF (docetaxel 35 mg/m^2^ days 1/15, cisplatin 40 mg/m^2^ days 1/15, 5FU 400 mg/m^2^ days 1–5, 15–19) as the first-line regimen. The third day after starting chemotherapy, she felt strong abdominal pain, and internal necrosis of lymph nodes around the primary lesion and free air in the abdominal cavity were found. Peritonitis was diagnosed due to a fistula formed from the lower thoracic ESCC to the peritoneum through lymph node metastases. She underwent emergency laparoscopic drainage, omental filling, and jejunostomy. Postoperatively, her general condition and inflammatory findings improved within 10 days, and she could continue intensive chemotherapy as scheduled.

**Discussion:**

Because of the risk of perforation and fistula in regimens that are expected to cause tumor shrinkage, careful observation may be required after starting chemotherapy.

**Conclusion:**

We report the first case of peritonitis caused by perforation through lymph node metastasis of thoracic esophageal cancer.

## 1 Introduction

Anatomically, the thoracic esophagus is surrounded by the aorta, pericardium, trachea, bronchi, lungs, and vertebrae, which are often invaded by advanced esophageal cancers. Even if the esophageal cancer is superficial, it tends to cause lymph node metastasis [1], [2], and in advanced cancer, multiple lymph node metastases are often observed. Esophageal fistula after treatment with chemoradiotherapy for locally advanced esophageal cancer is sometimes reported and becomes a severe adverse event for the patient [3], [4]. However, the occurrence of an esophageal fistula or perforation in a patient treated only by chemotherapy for advanced esophageal cancer is rare. In recent years, the response of a triplet regimen for advanced esophageal cancer has been reported [5], [6]. Here, we report the first case, to our knowledge, of peritonitis caused by perforation of lymph node metastases and formation of a fistula from the lower thoracic esophagus to the abdominal cavity after the initial administration of biweekly combined therapy of docetaxel, cisplatin, and 5FU (Bi-DCF) therapy for lower thoracic esophageal cancer.

## 2 Case report

**Fig. 1:**
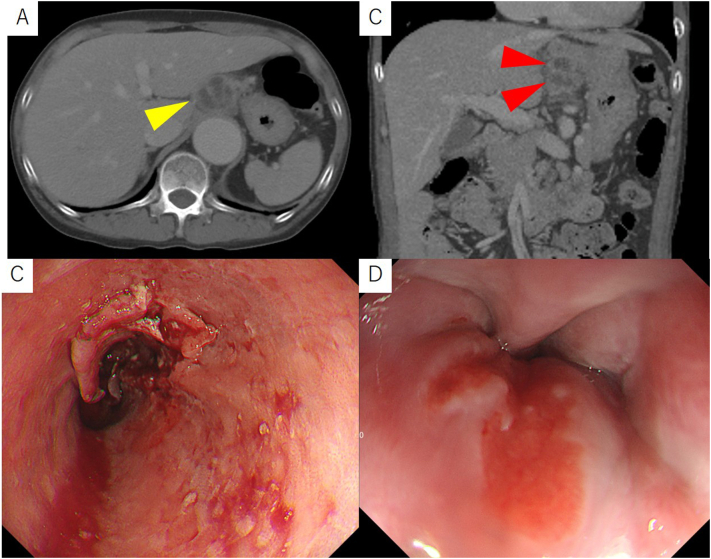
(A) Lymph node metastasis in lesser curvature side of stomach (yellow arrow head). (B) Lymph node metastases that forms a dumpling from around the esophagus to the lesser curvature of the stomach (red arrow head). (C) Primary lesion of lower thoracic esophageal squamous cell carcinoma. Type 3 tumor with stenosis was observed. (D) There was no tumor invasion to esophagogastric junction.

**Fig. 2:**
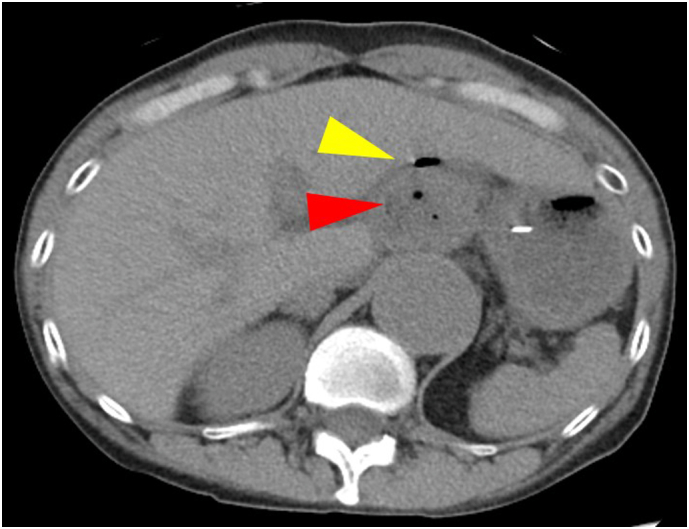
Computed tomography findings on the third day after induction of chemotherapy. Free air in the abdominal cavity (yellow arrowhead) and necrosis in lymph node metastasis in the lesser curvature side of the stomach (red arrowhead) were observed.

**Fig. 3:**
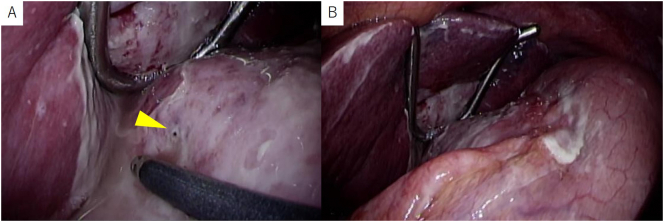
Intraoperative laparoscopic findings. (A) A small hole in the metastatic lymph node causing peritonitis was observed (yellow arrowhead). (B) No perforation of the stomach wall was observed. Inflammation around the lesser curvature of the stomach was prominent.

**Fig. 4:**
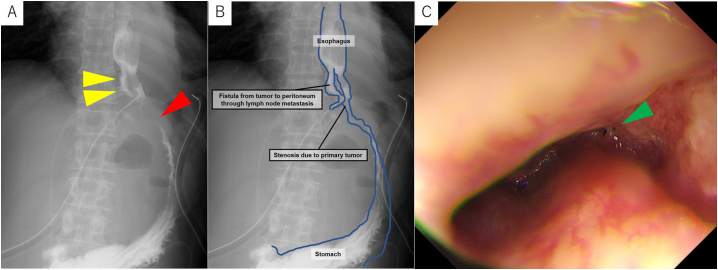
Postoperative upper gastrointestinal fluoroscopy and upper gastrointestinal endoscopy findings. (A) Fistula from the primary lesion to the peritoneum through lymph node metastases was observed (yellow arrowhead). The route flowing into stomach was different (red arrowhead). (B) Shamer of postoperative upper gastrointestinal fluoroscopy. (C) A fistula appears to connect to the lymph node metastases from the bottom of the ulcer in the primary lesion (green arrowhead).

A 46-year-old woman with no pertinent past history felt chest pain and consulted her home doctor. She was a smoker with 20 cigarettes per day and also a drinker with 2000 ml beer per day for 25 years. Enlargement of the mediastinum was noted on chest X-ray, and she was referred to our hospital for further evaluation and treatment. Contrast-enhanced computed tomography (CT) performed at her first visit showed a thoracoabdominal aortic aneurysm and wall thickening of the lower thoracic esophagus that was suspicious of esophageal cancer. In addition, dumpling-shaped swollen lymph nodes suspected of being lymph node metastases were present from the lower edge of the primary lesion in the lower mediastinum to around the lesser curvature of the stomach (Fig. 1A,B). Moreover, distant lymph node metastases around the abdominal aorta and liver metastasis were also observed. Esophagogastroduodenoscopy showed a circumferential type 3 tumor with stenosis in the lower thoracic esophagus, which led to dysphagia (Fig. 1C), and biopsy of the primary lesion showed squamous cell carcinoma. There was a distance of about 1 cm from the lower edge of the tumor to the esophagogastric junction (Fig. 1D). Her initial diagnosis was esophageal squamous cell carcinoma T3 N3 M1 (para-aortic lymph nodes and liver) Stage IVB according to the 8th edition of the UICC TNM classification. Considering the condition of her thoracic aortic aneurysm, it became a treatment strategy aiming at tumor shrinkage with intensive chemotherapy without radiation therapy. Therefore, Bi-DCF (docetaxel 35 mg/m^2^ on days 1 and 15, cisplatin 40 mg/m^2^ on days 1 and 15, and 5FU 400 mg/m^2^ on days 1–5 and 15–19) was started as the first-line regimen along with nutrition therapy using a small-diameter feeding tube. After the start of chemotherapy, her severe dysphagia improved and fluid intake became possible. On the 3rd day after the start of therapy, she complained of strong abdominal pain. An abdominal CT showed internal necrosis of the lymph nodes extending from the primary lesion to the lymph nodes around the lesser curvature of the gastric wall, and free air in the abdominal cavity just ventral to the lymph nodes was also pointed out (Fig. 2). Therefore, she was suspected of having peritonitis due to perforation of lymph node metastases through the primary lesion. She underwent emergency laparoscopic drainage, omental filling, and jejunostomy. Operative findings revealed a small hole in the lymph nodes around the lesser curvature of the stomach (Fig. 3A) and purulent ascites spread throughout the abdomen, but the gastric wall was intact (Fig. 3B). Postoperative upper gastrointestinal fluoroscopy revealed contrast medium flowing into the abdominal cavity (Fig. 4A, B), and upper gastrointestinal endoscopy confirmed a fistula from the esophagus to the lymph nodes (Fig. 4C). Cytopathology of the ascites collected during surgery showed no malignant cells. The patient was diagnosed as having peritonitis due to lymph nodes perforation from the primary tumor following intensive chemotherapy. After the surgery, her inflammatory findings improved without the formation of abscesses. Although several drain replacements were required for a while, intensive chemotherapy was continued as scheduled.

## 3 Discussion

To our knowledge, there have been no reports of thoracic esophageal cancer perforating into the abdominal cavity via lymph node metastases after chemotherapy as in this case, and this appears to be a very rare occurrence.

Esophageal cancer is one of the cancers that easily causes lymph node metastasis [1], [2]. In Japan, the naming and numbering of lymph nodes are defined in the Japanese Classification of Esophageal cancer, 11th Edition [7], [8]. In addition, lymph node groups according to the location of the tumor are defined by different susceptibility to metastasis. In lower thoracic esophageal cancer, metastasis is likely to occur from the lower thoracic paraesophageal lymph nodes to the lesser curvature lymph nodes along the left gastric artery, which are named #110, #20, #1, #3, and #7. In the present case, the lymph nodes that had metastasized from the primary lesion to lymph nodes #110, #1, and #3 were connected in a dumpling shape.

In recent years, a regimen combining docetaxel, cisplatin, and 5FU therapy (DCF) has been used not only for esophageal cancer but also for gastric cancer and head and neck cancer and is described in the NCCN guidelines. This triplet regimen has shown a high response rate and shown good clinical outcomes. In a recent phase 3 clinical trial, JCOG1109 NExT trial, conducted in Japan, DCF regimen has been shown to be optimal as neoadjuvant therapy for stage IB-III esophageal squamous cell carcinoma [9], [10]. The result of the trial showed that the DCF arm not only reduced perioperative complications but also showed better overall survival and progression free survival compared to the 5FU plus cisplatin (CF) arm and radiation with CT arm. On the other hand, grade 3–4 adverse events during adjuvant therapy were more common in DCF arm. Therefore, in this present case, we use the Bi-DCF regimen, which is more tolerable and has a higher response rate. Hara et al. reported that the DCF regimen was used as preoperative chemotherapy for esophageal cancer with a response rate of 64%, Grade 3/4 leukopenia of 45%, and neutropenia of 83% [5]. In our institution, Bi-DCF therapy as neoadjuvant chemotherapy for esophageal cancer has resulted in Grade 3/4 leukopenia of 12.5%, neutropenia of 31.4%, and no instances of renal dysfunction while maintaining a high response rate of 90.3% [6], [11].

The present patient had esophageal cancer with stenosis, but a thoracic aortic aneurysm was also present, and there was concern that the aneurysm would be exacerbated by radiation therapy. In addition, as the tumor shrinks, improvement of stenosis can be expected, so Bi-DCF therapy was selected, and nutritional management was performed simultaneously with a small-diameter feeding tube.

Gastrointestinal perforation during chemotherapy is occasionally found in unresected colorectal cancer, especially as a side effect of bevacizumab, a humanized monoclonal antibody, and occurs at rate of 1–2%. In the BRITE registry, 37 of 1953 metastatic colorectal patients suffered gastrointestinal perforation [12]. Esophageal cancer is close to surrounding organs and may form fistulas with surrounding organs as a result of chemoradiotherapy or radiotherapy. The incidence of esophageal perforation or fistula among patients with esophageal cancer treated with radiotherapy is around 5.6–33% [4]. Besides, gastrointestinal perforation following neoadjuvant chemotherapy for ESCC has also reported [13]. But it was the result of mucosal breakage of the gastrointestinal tract, which is caused by adverse event of chemotherapy, premedication of steroid and the absence of proton pump inhibitor. In our present case, there were no perforations in the gastric wall or duodenal wall, and it was considered that rapid tumor shrinkage after intensive chemotherapy formed a fistula from the bottom of the tumor ulcer to the lymph nodes.

We could find no report of thoracic esophageal cancer perforating the abdominal cavity via lymph node metastasis after the introduction of chemotherapy in our search of PubMed, and we consider this to be the first reported case of peritonitis caused by perforation through lymph node metastasis of thoracic esophageal cancer. In addition, cytopathology of the ascites collected during the operation showed no malignant findings, suggesting that the internal necrosis was caused by the markedly high response to Bi-DCF therapy. Following the results of clinical trials, intensive chemotherapy for the treatment of esophageal cancer is likely to expand, and meticulous attention is required after the introduction of chemotherapy.

This work was reported in line with the SCARE 2020 criteria [14].

## 4 Conclusion

We report the first case of peritonitis caused by perforation through lymph node metastasis of thoracic esophageal cancer. Because of the risk of perforation and fistula in regimens that are expected to cause tumor shrinkage, careful observation may be required after the start of chemotherapy.

## Abbreviations

ESCC: Esophageal squamous cell carcinoma
CT: Computed tomography
NCCN: National comprehensive cancer network

## Consent for publication

Written informed consent was obtained from the patient for publication of this case report and accompanying images. A copy of the written consent is available for review by the Editor-in-Chief of this journal on request.

## Data availability

All data generated or analyzed during this study are included in this published article.

## Provenance and peer review

Not commissioned, externally peer-reviewed.

## Ethical approval

This case study is exempt from ethical approval.

## Funding

This research did not receive any specific grant from funding agencies in the public, commercial, or not-for-profit sectors.

## Guarantor

Yoshihiro Tanaka, corresponding author of this article.

## Research registration number

N/A.

## CRediT authorship contribution statement

Study conception and design: TS and YT. Acquisition of data: TS, YT, YS, MF, IY and KY. Analysis and interpretation of data: TS and YT. Drafting of manuscript: TS and YT. Critical revision: TS, YT, and KY. Supervision: YT and KY.

## Declaration of competing interest

Dr. Yoshida reports receipt of grants, personal fees and non-financial support from EA Pharma Co., Ltd., Sanofi, Yakult Honsha Co., Ltd., Chugai Pharmaceutical Co., Ltd., Taiho Pharmaceutical Co., Ltd., Takeda Pharmaceutical Co., Ltd., Eli Lilly Japan K.K., Daiichi Sankyo Co., Ltd., Ono Pharmaceutical Co., Ltd., Merck Serono Co., Ltd., and Novartis Pharma K.K. and grants from Kyowa Hakko Kirin Co., Ltd. outside of the submitted work. Dr. Tanaka reports receipt of grants from Daiichi Sankyo Co., Ltd. outside of the submitted work. All of the other authors declare that they have no conflicts of interest.
